# The nervous system of *Isodiametra pulchra* (Acoela) with a discussion on the neuroanatomy of the Xenacoelomorpha and its evolutionary implications

**DOI:** 10.1186/1742-9994-9-27

**Published:** 2012-10-16

**Authors:** Johannes Georg Achatz, Pedro Martinez

**Affiliations:** 1 Department of Evolutionary Developmental Biology, University of Innsbruck, Technikerstrasse 25, 6020 Innsbruck, Austria; 2 Department of Genetics, University of Barcelona, Av. Diagonal, edifici annex, planta 2a, 08028 Barcelona, Spain; 3 Institució Catalana de Recerca i Estudis Avançats (ICREA), Passeig Lluís Companys 23, 08010, Barcelona, Spain

**Keywords:** Brain, Serotonin, FMRF, Tubulin, Evolution, Phylogeny

## Abstract

**Introduction:**

Acoels are microscopic marine worms that have become the focus of renewed debate and research due to their placement at the base of the Bilateria by molecular phylogenies. To date, *Isodiametra pulchra* is the most promising “model acoel” as it can be cultured and gene knockdown can be performed with double-stranded RNA. Despite its well-known morphology data on the nervous system are scarce. Therefore we examined this organ using various microscopic techniques, including histology, conventional histochemistry, electron microscopy, and immunocytochemistry in combination with CLSM and discuss our results in light of recently established phylogenies.

**Results:**

The nervous system of *Isodiametra pulchra* consists of a bilobed brain with a dorsal posterior commissure, a frontal ring and tracts, four pairs of longitudinal neurite bundles, as well as a supramuscular and submuscular plexus. Serotonin-like immunoreactivity (SLI) is displayed in parts of the brain, the longitudinal neurite bundles and a large part of the supramuscular plexus, while FMRFamide-like immunoreactivity (RFLI) is displayed in parts of the brain and a distinct set of neurons, the longitudinal neurite bundles and the submuscular plexus. Despite this overlap SLI and RFLI are never colocalized. Most remarkable though is the presence of a distinct functional neuro-muscular system consisting of the statocyst, tracts, motor neurons and inner muscles, as well as the presence of various muscles that differ with regard to their ultrastructure and innervation.

**Conclusions:**

The nervous system of *Isodiametra pulchra* consists of an insunk, bilobed brain, a peripheral part for perception and innervation of the smooth body-wall musculature as well as tracts and motor neurons that together with pseudostriated inner muscles are responsible for steering and quick movements. The insunk, bilobed brains with two to three commissures found in numerous acoels are homologous and evolved from a ring-commissural brain that was present in the stem species of acoelomorphs. The acoelomorph brain is bipartite, consisting of a *Six3/6*-dependend animal pole nervous system that persists throughout adulthood and an axial nervous system that does not develop by exhibiting a staggered pattern of conserved regulatory genes as in other bilaterians but by a nested pattern of these genes. This indicates that acoelomorphs stem from an ancestor with a simple brain or with a biphasic life cycle.

## Introduction

Acoels are microscopic, hermaphroditic and acoelomate worms that predominantly live in benthic marine habitats. Their relatively simple morphology but the high plasticity of their neuroanatomy was recognized early on [1]; however, there are some shared traits such as the possession of a peripheral plexus and 3–5 pairs of neurite bundles, which usually have a similar diameter and are distributed regularly spaced around the antero-posterior axis. The brain can be shaped like a ring, a barrel, or a bilobed mass with a complex connectivity of various neurites forming connectives and commissures [2-11]. Different parts of the nervous system have been revealed by immunocytochemistry, including those with serotonin-like immunoreactivity [3-10] and immunoreactivity against amines [4,7,11] and cholinergic parts by conventional histochemistry [8,12].

Since molecular phylogenetics revealed that the Acoela are not members of the Platyhelminthes but are rather the sister group to all other Bilateria [13-16] or nested at the base or within the Deuterostomia [17] research on these worms has been revived. Species on which the most work has been conducted are the convolutids *Convolutriloba longifissura*[8,18-22] and *Symsagittifera roscoffensis*[10,12,23-25] and the isodiametrid *Isodiametra pulchra*[26,27]. The latter lives in marine mud flats in Maine (USA) and measures about 1 mm in length. For the most part, specimens are translucent, feed on diatoms, lay 1–2 eggs per worm per day throughout the whole year and can be cultured in Petri dishes under laboratory conditions [26]. Besides the ease of culturing this species, the establishment of gene-knockdown with double-stranded RNA [26,27] makes *Isodiametra pulchra* a promising model system for the Acoela. However, despite the relatively detailed knowledge of its morphology [28-35], data on its nervous system are scarce. Therefore, we studied this organ using a set of complementary methods to give a detailed description, provide a basis for future studies investigating the effects of knockdown of genes involved in neurogenesis, and advance our understanding of the constraints on the species’ neuroanatomy.

## Results

### Acetylcholine

**Figure 1:**
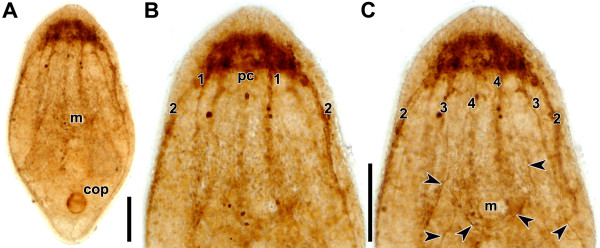
**Whole mount of mature specimen stained by direct-coloring method of Karnovsky and Roots**[36]**.A**. Entire specimen. **B**. Anterior half, dorsal side in focus. **C**. Anterior half, ventral side in focus. Arrowheads point to neurons in an anterior-posterior orientation and around the posterior rim of the mouth. Abbreviations: 1 dorsal neurite bundle; 2 lateral neurite bundle; 3 ventral neurite bundle; 4 medio-ventral neurite bundle; cop male copulatory organ; m mouth; pc dorsal posterior commissure. Scale bars: **A**, **B**, **C** 100 μm.

All specimens showed strong staining of the brain and the male copulatory organ (Figure 1A). The brain exhibits a commissure at the posterior rim or slightly posterior to the statocyst, which, in accordance with Raikova et al. [7], we term the dorsal posterior commissure. However, the area around the statocyst lacks any signal. Four pairs of neurite bundles are evident: a dorsal, a lateral, a ventral and a medio-ventral bundle (Figures 1B,C). There is an inconspicuous connection between the ventral and the medio-ventral pair approximately 25 μm behind the commissure. Distinct neurons extend neurites at various angles from anterior to posterior and around the posterior rim of the mouth (Figure 1C).

### Serotonin-like immunoreactivity (SLI)

**Figure 2:**
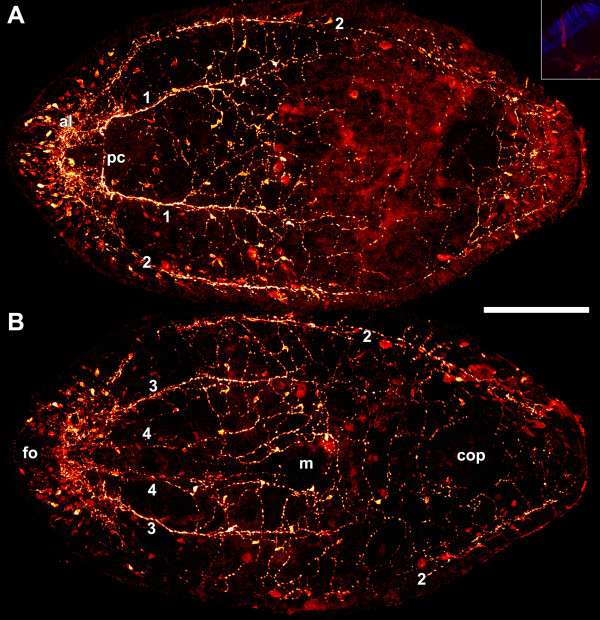
**Whole mount stained with polyclonal antibodies against serotonin.A**. Projection of dorsal sections. **B**. Projection of ventral sections. Inset: Projection of body wall showing cilia of epidermal cells in blue and receptor cell with single cilium with SLI in red. Note that the soma of the cell was out of the section plane and its intensity is therefore weaker. Abbreviations: 1 dorsal neurite bundle; 2 lateral neurite bundle; 3 ventral neurite bundle; 4 medio-ventral neurite bundle; al anterior lobe; cop male copulatory organ; fo frontal organ; m mouth; pc dorsal posterior commissure. Scale bar: 100 μm.

**Figure 3:**
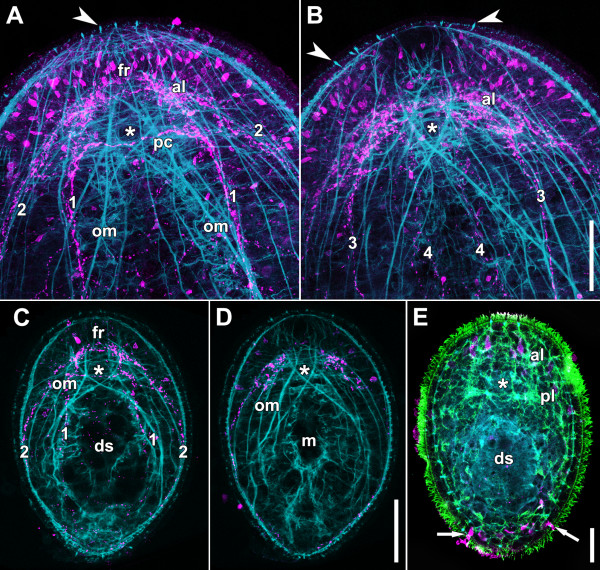
**Whole mounts stained with polyclonal antibodies against serotonin (magenta: A, B, C, D, E), tyrosinated tubulin (green: E), and fluorophore-tagged phalloidin (cyan).** Asterisks mark position of statocyst. **A**. Anterior end of adult specimen, projection of dorsal sections. The dorsal body-wall musculature has been omitted for clarity. Arrowhead points to swallow’s nest receptor cell. Note the position of the X-muscle ventral to the dorsal posterior commissure. **B**. Anterior end of adult specimen, projection of ventral sections. Arrowheads point to swallow’s nest receptor cells. Note the position of the X-muscle ventral to the statocyst. **C**. Dorsal projection of juvenile. Note the position of the X-muscle ventral to the dorsal posterior commissure. **D**. Ventral projection of juvenile. Note the position of the X-muscle ventral to the statocyst. **E**. Dorsal projection of juvenile. Arrows point to gland cells at posterior tip. Abbreviations: 1 dorsal neurite bundle; 2 lateral neurite bundle; 3 ventral neurite bundle; 4 medio-ventral neurite bundle; al anterior lobe; ds digestive syncytium; fr frontal ring; m mouth; om oblique inner muscle; pc dorsal posterior commissure; pl posterior lobe. Scale bars: **A**, **B** 50 μm; **C**, **D** 50 μm; **E** 20 μm.

Serotonin-like immunoreactivity (SLI) is present in a peripheral plexus with somata and neurites that pervade the periphery of the entire body plus an internal mass of neurites in the brain (Figures 2A,B). Somata are especially numerous at the anterior end except in the area of the frontal organ, projecting neurites into the brain that measure up to 20 μm in length. The majority of somata at the anterior end lie below the body-wall musculature, whereas in the rest of the body they are located peripheral to the body-wall musculature (Figures 3A,B). In many cases a single stained cilium at the apical tip of these somata is apparent (Figure 2, inset). The peripheral plexus condenses into three major pairs of longitudinal neurite bundles that lie beneath the body-wall musculature: a dorsal, a lateral and a ventral pair, plus one pair of minor medio-ventral longitudinal bundles, all extending frontally and terminating in the brain (Figures 2A,B, 3A,B). At the most frontal tip, neurites are often found to form an inconspicuous frontal ring (Figures 3A,C). The ventral and dorsal neurite bundles extend to the level of the mouth, where they merge into the plexus around the mouth or the area on the dorsal side of the mouth, respectively. The lateral bundles extend all the way to the posterior end, where they merge with the plexus approximately 25 μm away from the posterior tip. The medio-ventral bundles split about 100 μm posterior to the statocyst, merging into the plexus around the mouth, with the innermost neurites bending around the mouth. The areas noted above are the most conspicuous parts of the basiepidermal plexus (Figures 2A,B).

**Figure 4:**
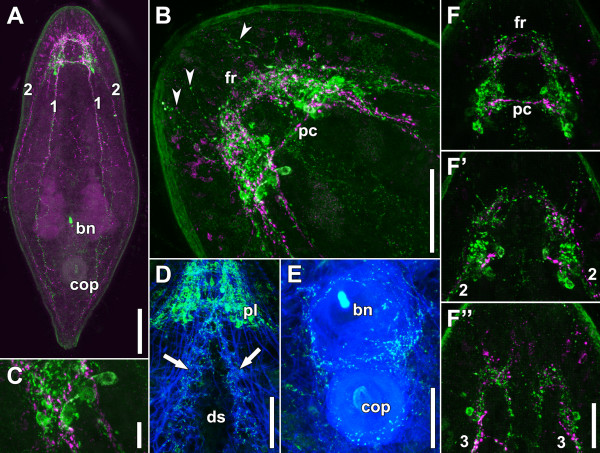
**Whole mounts of adult specimens stained with antibodies against FMRFamide (green: A, B, C, F, F’, F”), serotonin (monoclonal; magenta: A, B, C, F, F’, F”), and fluorophore-tagged phalloidin (blue; D, E).A**. Dorsal side of entire specimen. **B**. Anterior end. Arrows point to lateral varicosities of neurites that probably connect the lobes with receptor cells in the epidermis. **C**. Detail of B. **D**. Anterior half of a specimen showing RFL immunoreactive parts of the brain and neurites surrounding the dorso-ventral muscles of the ventral groove. **E**. Musculature of copulatory organs innervated by RFL immunoreactive neurites. **F**. Dorsal projection of brain. **F’**. Central projection of brain. **F”**. Ventral projection of brain. Abbreviations: 1 dorsal neurite bundle; 2 lateral neurite bundle; 3 ventral neurite bundle; bn bursal nozzle; cop male copulatory organ; ds digestive syncytium; fr frontal ring; pc dorsal posterior commissure. Scale bars: **A** 100 μm; **B** 50 μm; **C** 10 μm; **D** 50 μm; E 50 μm; F-F” 25 μm.

**Figure 5:**
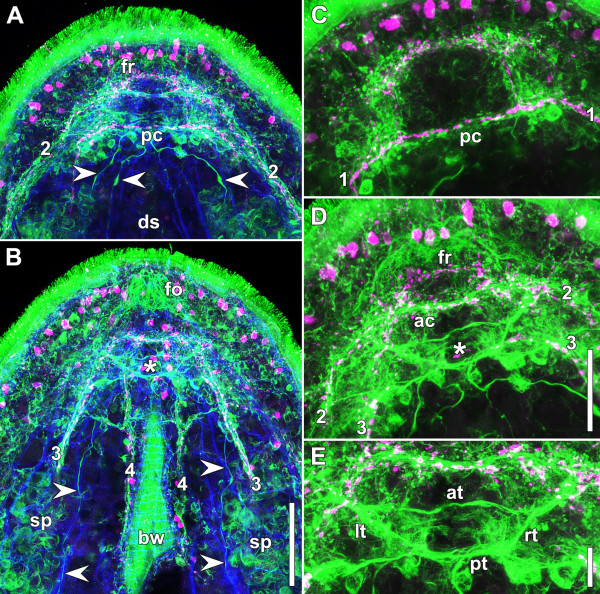
**Whole mount stained with antibodies against tyrosinated tubulin (green: A, B, C, D, E), serotonin (polyclonal; magenta: A, B, C, D, E), and fluorophore-tagged phalloidin (blue: A, B).A**. Projection of central sections. Arrowheads point to neurites that follow distinct inner muscles. **B**. Projection of ventral sections. Arrowheads point to neurites that follow distinct inner muscles. Asterisk marks position of statocyst. **C**. Projection of dorsal sections. **D**. Projection of central sections. Asterisk marks position of statocyst. **E**. Magnification of D. Abbreviations: 1 dorsal neurite bundle; 2 lateral neurite bundle; 3 ventral neurite bundle; 4 medio-ventral neurite bundle; at anterior tract; bw body wall; ds digestive syncytium; fo frontal organ; fr frontal ring; lt left tract; pc dorsal posterior commissure; pt posterior tract; rt right tract; sp spermatids and sperm. Scale bars: **A**, **B** 50 μm; **C**, **D** 30 μm; **E** 10 μm.

There are three conspicuous structures in the brain: a frontal nerve ring, a paired dense aggregation of neurites located latero-caudally to the frontal ring, which will subsequently be termed the anterior lobes in accordance with Smith and Bush [29], and the dorsal posterior commissure (Figures 2A,B, 3A,B). This commissure lies on top of the intercept point of the crossing parenchymal muscles (terminology in accordance with [32]), which are among the most striking inner muscles (terminology in accordance with [31]) and will subsequently be called X-muscles (Figures 3A,B,C,D). When entering the brain, the dorsal neurite bundles bend slightly ventrally and seem to disintegrate into paired areas of high connectivity, which are far less apparent than the anterior lobes but which nevertheless will subsequently be termed the posterior lobes in accordance with Smith and Bush [29]. However, two tracts are consistent and strong enough to follow: one continues further anteriorly, connecting to the corresponding anterior lobe, and the other bends towards the midline, becoming part of the posterior commissure. About 12 μm towards the midline from where the dorsal neurite bundle “splits”, a tract extends directly ahead to the anterior lobe, together with the dorsal side of the frontal ring and the posterior commissure forming a trapezoid structure on top of the statocyst (Figures 2A, 3A, 4A,B,F, 5C). The lateral neurite bundle can be followed all the way to the anterior lobe, and of the many connections to the adjacent nerve cords the following are apparent: one to the posterior lobe, located at the level of the dorsal posterior commissure, and one to the ventral neurite bundle, occurring approximately 15 μm posterior to this commissure. The ventral neurite bundles enter the posterior lobe and extend to the lateral sides of the commissure. However, many bundles of neurites extend towards the anterior lobe and there is a conspicuous connection with the medio-ventral neurite bundles approximately 10 μm posterior to the dorsal posterior commissure. The medio-ventral neurite bundles pass the posterior lobes and terminate straight in the anterior lobes (Figure 2B).

### FMRFamide-related immunoreactivity (RFLI)

FMRFamide-like immunoreactivity is present in a submuscular plexus with somata and neurites in the entire periphery of the body and an internal mass of somata and neurites in the brain (Figures 4A, B). The FMRFamide-like immunoreactivity follows the same pattern as SLI with slight deviations, although no colocalization was observed (Figures 4A,B,C,F-F”). There are four pairs of neurite bundles: a dorsal, a lateral, a ventral and a medio-ventral bundle. All bundles emanate from the posterior lobes except for the medio-ventral bundles, which emanate from the anterior lobe. The dorsal bundles extend to the level of the male copulatory organ, where they fan out towards the midline and the lateral neurite bundles, while the lateral neurite bundles extend to the posterior end and merge with the plexus in this area about 25 μm away from the posterior tip (Figure 4A). The ventral bundles extend to the level of the bursal nozzle, where they fan out to constitute a dense net that innervates the copulatory organs (Figure 4E). The medio-ventral neurite bundles extend to the mouth, possibly encircling it. There are two prominent rows of dorso-ventral muscles along the ventral groove, which are innervated by FMRFamide-related immunoreactive neurites (Figure 4D).

Concordant with SLI, RFLI comprises the dorsal posterior commissure and the frontal nerve ring in the brain (Figures 4A,B,F). Contrary to SLI, there is no clear separation between the anterior and posterior lobes but instead there are rather two paired lobes that reach from the lateral edges of the frontal ring to a short distance posterior to the dorsal commissure (Figures 4B,F−F”). The number and density of neurites, however, is higher in the posterior areas, which, in accordance with the terminology of SLI are termed the posterior lobes (Figures 4F−F”). The number and position of distinct neurons within the brain that show RFLI was fixed in all specimens examined. There is a pair of bipolar neurons lateral to the ventral neurite bundles at the level of the dorsal posterior commissure (Figure 4F”) and two pairs of unipolar neurons between the lobes: the ventral one at the level of the anterior rim of the dorsal posterior commissure and the dorsal one approximately 10 μm in front of it (Figures 4F and F’). Three to five pairs of unipolar neurons occur in the posterior region of the posterior lobes, approximately between the level of the lateral and dorsal neurite bundles (Figures 4B,C, 4F, F’). The staining intensity of these neurons varies greatly and weakly stained cells can be obscured merely by the density of neurites in this area. Neurites with RFL immunoreactive varicosities that extend from the lateral sides of the lobes to the lateral sides of the anterior tip of the animals were apparent in all specimens (Figures 4B,F’). No stomatogastric RFLI was detected.

### Tubulin

**Figure 6:**
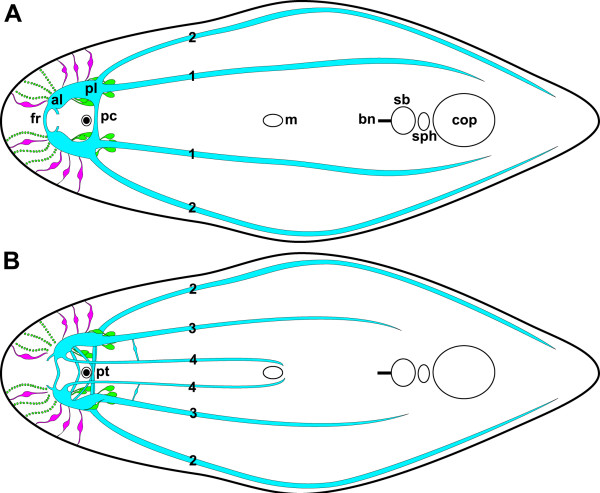
**Schematic drawing of the nervous system of *I. pulchra *(green: RFLI; magenta: SLI; cyan: central nervous system).A**. Dorsal view. **B**. Venral view. Abbreviations: 1 dorsal neurite bundle; 2 lateral neurite bundle; 3 ventral neurite bundle; 4 medio-ventral neurite bundle; al anterior lobe; bn bursal nozzle; cop male copulatory organ; fr frontal ring; m mouth; pc dorsal posterior commissure; pl posterior lobe; pt posterior tract; sb seminal bursa; sph sphincter.

Even though tubulin is present in many structures of the body, for example in the axonemes of spermatozoa and cilia of epidermal cells, as well as in the cell wall of frontal gland cells, the nervous system can be revealed with high clarity using specific anti-tubulin antibodies (Figures 5A,B). All structures detected by SLI and RFLI appear even more pronounced with the monoclonal antibody against tyrosinated tubulin, namely all four pairs of neurite bundles (Figures 5A,B,C,D, 6), the anterior and posterior lobes (Figures 5A,C, 6), the dorsal posterior commissure (Figures 5A,C, 6), and the frontal ring (Figures 5A,D, 6). The latter structure, however, does not appear as a homogeneous ring but as a strong bundle of neurites or a tract on the ventral side and a weaker part on the dorsal side (Figure 5D). Additionally, anti-tubulin staining reveals tracts around the statocyst and neurons, the somata of which lie at the level of the dorsal posterior commissure or slightly posterior to it, each one extending a neurite along a distinct inner muscle (Figures 5A,B,D). The tracts and neurons form a pattern that was highly conserved among all studied specimens. The anterior tract connects the anterior lobes, the posterior tract the posterior lobes, and two paired crossover tracts connect one anterior lobe with the posterior lobe of the opposite side via the posterior tract. The roots of the anterior tracts and the crossover tracts have a common origin in the anterior lobes (Figure 5E).

### Histology and electron microscopy

**Figure 7:**
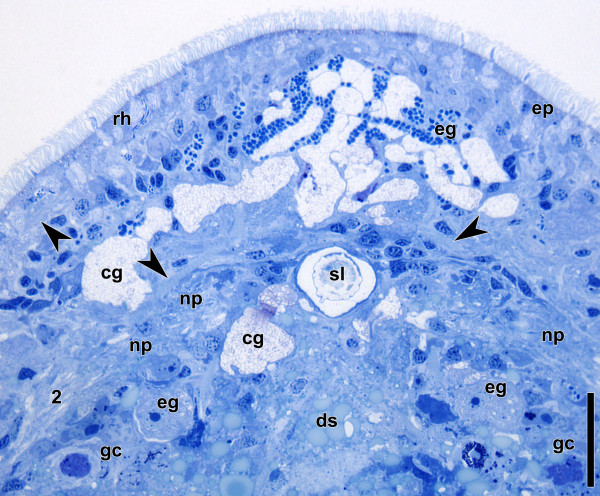
**Horizontal semithin section through anterior end of adult specimen stained with Richardson's.** Arrowheads point to muscles. Note nuclei around the statocyst and the anterior neuropil, pseudostriation in highlighted inner muscles, and metaphase chromosomes in germ cells. Abbreviations: 2 lateral neurite bundle; cg cyanophilic gland cells; ds digestive syncytium; eg eosinophilic gland cells; ep epidermis; gc germ cells; np neuropil; rh rhabdoids; sl statolith. Scale bar: 25 μm.

**Figure 8:**
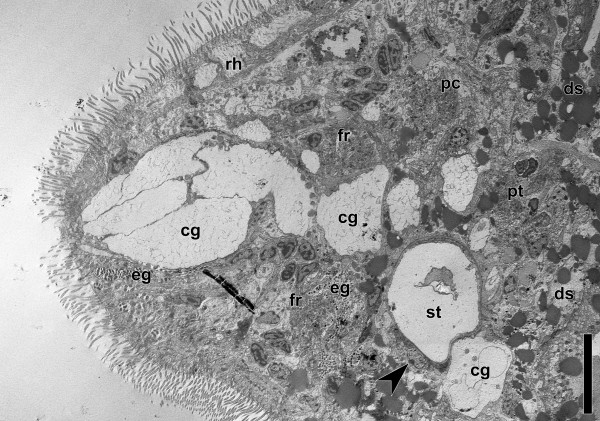
**Electron micrograph of sagittal section through anterior end of adult specimen.** Arrowhead points to ventral polar cell. Note various tissues extending through brain and lipid droplets ventral to statocyst. Abbreviations: cg cyanophilic gland cells; ds digestive syncytium; eg eosinophilic gland cells; fr frontal ring; pc dorsal posterior commissure; pt posterior tract; rh rhabdoids; st statocyst. Scale bar: 10 μm.

**Figure 9:**
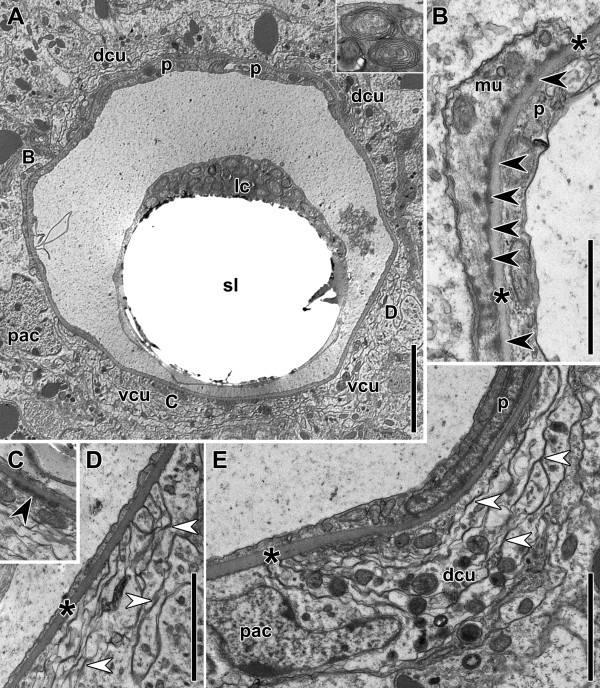
**Electron micrographs of cross sections through the statocyst of an adult specimen.** Asterisks mark the ECM of the statocyst. **A**. Cross section through the statocyst. Inset: multilaminar bodies of the lithocyte. **B**. Magnification of A. Black arrowheads mark dense plucks of muscle cell attached to statocyst capsule. **C**. Magnification of A showing a dense junction of cushion with statocyst capsule. **D**. Magnification of A showing part of the ventral cushion. White arrowheads point to synapse-like structures. **E**. Dorso-lateral cushion. Picture is rotated clock-wise; position of parietal cell nucleus is usually dorso-lateral. White arrowheads point to synapse-like structures. Abbreviations: dcu dorsal nerve cushion; mu muscle; lc lithocyte; pac parenchymal cell; p parietal cell; sl statolith; vc ventral nerve cushion. Scale bars: **A** 5 μm; **B** 1 μm; **C**, **D**, **E** 2 μm.

**Figure 10:**
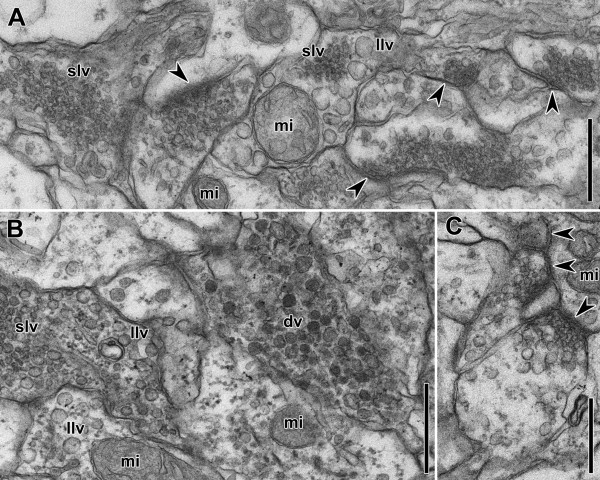
**Electron micrographs of horizontal sections through the brain of an adult specimen.** Arrowheads point to synapses. **A**, **B**. Area of neuropil showing various vesicles and synapses. **C**. Synapses. Abbreviations: dv dense vesicles; llv large lucent vesicles; mi mitochondrion; slv small lucent vesicles. Scale bars: **A**, **B**, **C** 500 nm.

The brain of *Isodiametra pulchra* is not demarcated with an extracellular matrix from non-nervous tissue and pervaded by frontal gland cells, inner muscles, extensions of the digestive syncytium, and peripheral parenchyma cells (Figures 7, 8). The neuropil is compact and there are accumulations of neurons around the statocyst and in the periphery of the anterior and the posterior lobe. However, they do not form a clear rind around these structures (Figure 7). The statocyst is constituted by a central lithocyte containing the statolith and two lining parietal cells (Figures 7, 8, 9A). The lithocyte contains numerous multilaminar bodies (Figure 9A) and a lens-like structure that is made up of thin tubules on the ventral side. On the ventral side of the statocyst lies a neuron termed the ventral polar cell in accordance with Ferrero ( [37]; Figure 8). There is also a so-called ventral nerve cushion surrounding the statocyst and two dorso-lateral nerve cushions in the area of the nuclei of the parietal cells. No synaptic contacts between neurons and the statocyst have been found, but there are large dense contacts between the cells of the cushions and the ECM of the capsule (Figure 9C), and some muscle fibres attached to the ECM of the capsule with button-like insertions (Figure 8B). There are many contacts between the membranes of the cushion that are reminiscent of synapses (Figures 8D,E), but synaptic vesicles are absent. In other parts of the brain, small clear vesicles (20–40 μm; Figures 10A,B), large lucent vesicles (~100 μm; Figures 10A,B), dense vesicles (~100 μm; Figure 1B) and dense core vesicles (~90 μm) are present. Large lucent vesicles were found together with small clear vesicles, but never with large dense vesicles in the same cell (Figures 10A,B). Synapses are omnipresent in the neuropil but the density of these structures is highest in the anterior lobe. All synapses found were unidirectional and most form dyad, triad, or tetrad sites (Figures 10A,B,C).

### Development

**Figure 11:**
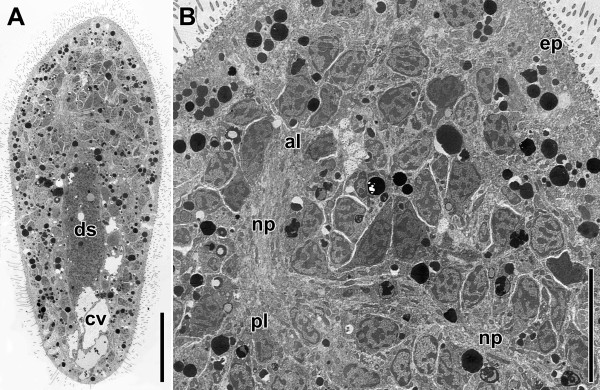
**Electron micrographs of a horizontal section through a juvenile specimen.A**. Whole specimen. **B**. Anterior end. Abbreviations: al anterior lobe; cv chordoid vacuole; ds digestive syncytium; ep epidermis; np neuropil; pl posterior lobe. Scale bars: **A** 25 μm; **B** 10 μm.

The gross anatomy of the nervous system of adults is already present in hatchlings and juveniles. The frontal ring, the anterior and posterior lobes, the posterior commissure, the four pairs of neurite bundles, the distinct pattern exhibited by the posterior commissure, the tracts close to the statocyst and the two pairs of X-muscles and oblique muscles are all present (Figures 3C,D,E, 11A,B). The statocyst, though, does not seem to be fully developed at this point. The lithocyte does not contain the large numbers of multilaminar bodies, the statolith is not yet present and the space between the lithocyte and the parietal cells is so limited that the lithocyte is barely able to float.

## Discussion

### Terminology

To readers used to the terminology of earlier work on the nervous systems of free-living flatworms some terms might be unfamiliar, however, given that comparisons of nervous systems between phyla have increased in recent years we decided to use more accurate and up-to-date terms, following the glossary for invertebrate neuroanatomy by Richter et al. [38]. The most striking replacement is the usage of neurite bundle instead of nerve cord. Concerning the latter it is noteworthy that a nerve cord can either be a medullary cord or a neurite bundle. The former is signified by a longitudinally extending central neuropil surrounded by a cell cortex consisting of neuronal somata distributed along its entire length, whereas neurite bundles do not show such a demarcation [38]. As the somata of neurons are distributed along the “nerve cords” but clearly do not form any kind of a cortex in acoelomorphs, the latter term must be used (see [10] for the most accurate study on this issue).

More difficult is the assessment of the presence of connectives in acoels. In the case of *I. pulchra* the longitudinal neurite bundles extending along the body disintegrate into the posterior or anterior lobe, respectively, and there are no distinct longitudinal bundles of neurites in the brain, therefore there is no need to use this term. However, such longitudinal neurite bundles are apparent in larger species [7,12]. Consequently the critical point would be if these bundles connect distinct ganglia. In a brain that lacks ECM and is surrounded and pervaded by non-neuronal tissue it is naturally difficult to find “distinct” units and consequently no such ganglia have been described using light microscopy. Nevertheless, the presence of two to three commissures, anterior and posterior lobes and the close spatial relation between distinct sets of cells revealed with RFLI and a distinct commissure indicate the subdivision of these brains into separate compartments and in our view justify the usage of this term in the publications with which we compare our results with further below.

Recently, an additional term has been introduced to avoid the question of wether acoels do have a brain, namely the “statocyst ganglion”. We found the highest density of synapses in the anterior lobes and these lobes are obviously not part of the nervous tissue surrounding the statocyst. Therefore, equating the terms brain and statocyst ganglion would exclude a large part of the connectivity and integration of cybernetic input from the latter. Consequently, the term “statocyst ganglion” [10] should only be employed to designate the nervous tissue in the vicinity of the statocyst or that has a functional relation with it. In fact, this definition fits very well, as an earlier term used to signify the nervous tissue surrounding the statocyst, the endonal brain, should be abandoned (personal communication Olga I. Raikova). The term endonal brain was introduced based on the assumption that neurons surrounding the statocyst in acoel and spiralian brains are homologous. However, based on developmental studies [39,40], structural properties (no SLI and RFLI in Acoelomorpha unlike in Platyhelminthes [7,41]), and phylogenetic considerations (see background), it can be concluded that these assumptions were wrong.

### Comparisons within the Acoela

By using antibodies against distinct transmitters or neuropeptides only distinct subsets of the nervous system are revealed [7]; however, comparison of patterns derived from the same antibody among related taxa should be valid. In this respect one drawback in comparing patterns of *I. pulchra* with acoel taxa is that there is no species of the “natural” Isodiametridae for which there are data available (see Figure 8 in [42]). Nevertheless, with regards to SLI, the pattern in *I. pulchra* is most reminiscent of *Avagina incola*[3,5,6].

The presence of a dorsal posterior commissure that is located in a similar position relative to the statocyst and with a similar pattern of connectivity to other parts of the brain in *Avagina incola*, childiids [5-7] and convolutids [8,10,11] is remarkable. Taking into account the position of RFL immunoreactive neurons in our material and in convolutids [11], together with the position of GYIRF immunoreactive neurons in childiids [7], the homology of this commissure cannot really be questioned. Even the frontal commissure, which forms a ring-like structure due to an additional dorsal arc in *I. pulchra*, is present in a relatively ventral position in all the aforementioned taxa. There are grounds for proposing that the frontal commissure and the dorsal posterior commissure are homologous in Isodiametridae and Aberrantospermata. Similarly, the dorsal frontal commissure, the dorsal anterior commissure and the dorsal posterior commissure in childiids (terminology of [7]) are homologous to the anterior commissures c1, c2, and c3 in *Symsagittifera roscoffensis*[10-12]. The dorsal anterior commissure could be a synapomorphy of the Aberrantospermata, but more research is needed on this issue. Nevertheless, the terminology of Raikova et al. [7] should be followed consistently in future studies on the nervous system of acoels whenever possible.

A conundrum in doing so in our case has been the denomination of the longitudinal neurite bundles. While dorsal, lateral, ventral and medio-ventral describe their position within the body accurately they seem to correspond, respectively, to the dorsal, dorso-lateral, ventro-lateral and ventral neurite bundles in childiids [7]. Although more data on other taxa are needed to resolve this issue, it is tempting to speculate that in comparison with childiids, *I. pulchra* has shifted the body wall towards the ventral side to enable the formation of a ventral groove, while convolutids seem to have done the opposite, namely widening the ventral side to cover the whole ventral surface, possibly to enable the capture of larger prey.

Also similar to other taxa are the distinct neurons that are located below the body-wall musculature at the anterior end (we wish to avoid the term sensilla because it has been coined for arthropod sensory structures that consist of a hair or pore in association with two receptor cells), which also occur in *Avagina incola* and childiids [3-6], as well as in convolutids [3,11].

The most striking peculiarity of *Isodiametra pulchra* is that the brain is devoid of any SL immunoreactive somata, a fact that has not been reported for any other species within the Acoela. We cannot explain this difference, but as we found the same result using two different antibodies we think that there is strong support for this conclusion. Here, it should be noted that the monoclonal and polyclonal antibodies gave identical results concerning the structures that were immunoreactive (compare Figures 2, 3 and 5 for the polyclonal antibody with Figure 4 for the monoclonal antibody); however, the signal of the monoclonal antibody was naturally weaker as only one specific epitope is recognized by monoclonal antibodies in comparison with many epitopes (and consequently fluorophore-tagged antibodies that will be bound to a structure) by polyclonal antibodies.

The presence of SLI in the cilia of receptor cells (see inset in Figure 2) seems unconventional and we are aware that there is no biological explanation for this. However, in investigations using EM we found amidergic vesicles in the vicinity of the ciliary rootlets and speculate that their content is not perfectly fixed by paraformaldehyde and partly diffuses into the cilium after Triton-X treatment (personal communication Willi Salvenmoser). This speculation is further corroborated by the diffuse SLI of the somata. Additionally, we found the same and even more distinct SLI in the cilia of receptor cells of other acoels (personal unpublished observations), and they are also present in other flatworms (personal communication Willi Salvenmoser). Consequently we interpret the SLI in single cilia of receptor cells as an artifact that, together with the position of the serotonin-like immunoreactive plexus peripheral to the body-wall musculature, allows us to argue that the SL immunoreactive nervous system comprises part of but not the entire sensory nervous system. A very prominent type of receptor cell that can be revealed with fluorophore-tagged phalloidin is the so-called swallow’s nest receptor cell ( [33]; Figures 3A,B); in none of our double-labeling experiments did we observe such receptor cells to have any connection with the SL immunoreactive plexus. A thorough investigation of a hatchling of *Symsagittifera roscoffensis* using electron microscopy [10] showed that all receptor cell types are distributed in distinct regions of the body and that contrary to *Isodiametra pulchra* the swallow’s nest receptor cells are not distributed in the frontal and caudal tip of the body [33] but in three paired rows, in parallel with the longitudinal neurite bundles.

Three conclusions can be drawn from these data:

First, only one type or subset of types of the present receptor cells use serotonin as the prevalent transmitter, and consequently only a subset of the sensory plexus is revealed with antibodies against this amine. With antibodies that recognize all transmitters present in all types of receptor cells, it is most probable that a plexus with the same intensity and maybe an even higher density than that at the level of the mouth revealed with the antibodies against serotonin would become apparent.

Second, the stronger immunoreactivity on the dorsal side of childiids [7] and convolutids [8,11] is probably due to a higher density of serotonin-like immunoreactive receptor cells on the dorsal side of these animals.

Third, assumptions about the evolution of the nervous system should not be drawn from an antibody staining against serotonin alone, as differences between taxa could reflect adaptations to different lifestyles and correlated body forms.

While discussing the serotonin-like immunoreactive nervous system, it should be noted that in contrast to *I. pulchra*, the plexus in childiids and convolutids is positioned below the body-wall musculature, a fact that might be due to the position of the epidermal somata beneath the body-wall musculature [9], or to an inversion of the different layers of the body-wall musculature [43].

The absence of RFamide-like immunoreactivity in connection with the mouth and gut is consistent with results from other acoels [7].

Our results from histology and electron microscopy are in keeping with previous studies. However, it must be stressed that we only made partial serial sections in the area of the statocyst in the orientation of the three body axes and therefore may have missed various structures that have been reported earlier. In contrast to the accurate studies on the central and peripheral nervous system conducted by Bedini and Lanfranchi [44], we did not detect presumptive glial cells or electron-dense vesicles mixed with small clear vesicles, and as we did not investigate the peripheral plexus we cannot verify the presence of symmetrical and electrical synapses. In agreement with the former authors, we found small clear vesicles to be the most abundant in the central nervous system, and in combination with the pattern of acetylcholine conclude that these are cholinergic vesicles.

All our findings on the statocyst are consistent with the description of this organ in *Symsagittifera psammophila*[37]. However, we were unable to determine the exact pattern of muscles that insert on the statocyst. In line with earlier claims [43] we suspect that this pattern might be a valuable character with which to infer relationships among acoels. The same applies to the position and numbers of nerve cushions (sensu [37]) on the statocyst. Whereas Ferrero [37] described only a ventral cushion in *S. psammophila*, we found an additional pair on the dorso-lateral sides of the statocyst.

With respect to development, we can only state that the general pattern of the central nervous system is present when animals hatch. However, similar to *S. psammophila*, the statocyst is not completely mature at that time. No clear results could be gathered with the antibodies used in this study. Antibodies against serotonin stained gland cells at the posterior end of the animals (Figure 3E), which disappeared shortly after hatching, and antibodies against FMRFamide-related peptides produced too much background. Moreover, embryos stained with antibodies against tyrosinated tubulin revealed too many structures in close vicinity to each other to provide us with a clear picture. To follow the development of the nervous system, new antibodies will be required.

### Neuroanatomy of the Acoelomorpha

**Figure 12:**
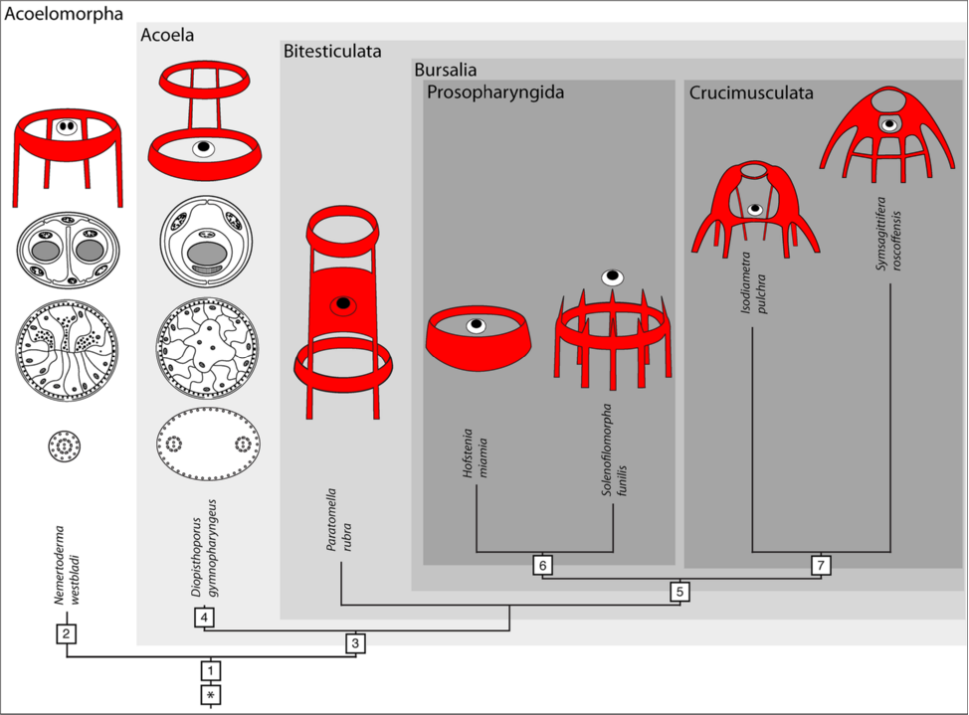
**Phylogeny of depicted acoelomorphs amended with schemes of corresponding nervous system (red), statocysts, digestive tracts and spermatozoa (from top to bottom).** (*) Hypothetical ancestor either with a bipartite brain that was not crossed by the alimentary tract and not staggered into forebrain, midbrain and hindbrain along the A-P axis but into anterior pole and axial nervous system or with a biphasic life cycle and an actively swimming and feeding larva, acoelomorphs being progenetic descendants. (1) Ring-commissure and small number of posterior neurite bundles. Adaptations to interstitial lifestyle: statocyst, frontal glands, multiciliary epidermis with special rootlet system and stepped tips of cilia, copulation. (2) Statocyst with two statoliths. Adaptation to internal fertilization: cork-screw-shaped sperm. (3) Nervous system looses basiepidermal position and tendency to develop an additional anterior ring commissure. Statocyst with one statolith, digestive system with unpolarized cells. Adaptation to internal fertilization: biflagellate sperm. (4) Posterior pharynx. (5) Female accessory organs. (6) Subterminal anterior pharynx. (7) Circular pattern of brain is abandoned and results in bilobed brains with one to three commissures. Branch-lengths estimated after [14,17], schemes of sperm, digestive tracts and statocysts from [9,45], pattern of nervous system from the following sources: *N. westbladi*[47]; *D. gymnopharyngeus*[48]; *P. rubra*[49]; *H. miamia*[50]; *S. funilis*[51]; *I. pulchra* [this paper]; *S. roscoffensis*[10-12].

As argued above the bilobed brains with a dorsal posterior commissure found in *Isodiametra pulchra*, childiids and convolutids show crucial shared traits and should therefore be considered homologous. However, all these taxa belong to the clade Crucimusculata (acoels with ventral crossover muscles and wrapping cells [42,45]), the members of which are relatively divergent [42]. Information on species in other clades has primarily been deduced from investigations of histological serial sections, which primarily focused on copulatory organs and were obviously problematic in animals with a well-developed frontal organ (see [46]). Nevertheless, with the updated phylogeny of acoels [42] and nemertodermatids [14], as well as the certainty that both together constitute the monophyletic clade Acoelomorpha [15,17], the evolution of the nervous system in these peculiar worms can be traced with some accuracy (see Figure 12).

The sistergroup of the Crucimusculata is the Prosopharyngida, which comprises the Hallangidae, Hofsteniidae and Solenofilomorphidae. Interestingly, the latter do not have a bilobed brain but one to three ring commissures in the vicinity of the statocyst and eight longitudinal neurite bundles (Figure 12; [51]). The nervous system is positioned below the body-wall musculature and some of the neurite bundles are closely associated with the epidermis (the somata of the epidermis are also positioned below the body-wall musculature) whereas others, especially the dorsolateral and ventrolateral bundles, tend to be larger and clearly separated from the epidermis [51]. Interestingly, in the sistergroup of the Solenofilomophidae, the Hofsteniidae, the relationship between the epidermis and nervous system is even more variable. In *Hofsteniola pardii* the nervous system consists of a basiepidermal plexus, in *Marcusiola tinga* the nervous system lies subepidermally but the dorsal neurite bundles are in a basiepidermal position, whereas in the most accurately investigated species, *Hofstenia atroviridis* and *Hofstenia miamia*, the entire nervous system is positioned below the epidermis [52]. Besides the accumulation of a few neurons around the statocyst the latter two species have a ring or a cylinder of nervous tissue that completely encircles the body in the region of the statocyst. This cylinder is thickest on the dorsal side, gradually becomes thinner towards the ventral side and comprises neuropil and somata, many of which are positioned below the body-wall musculature (Figure 12; [50,53]).

Crucimusculata and Prosopharyngida are united in the clade Bursalia with the Paratomellidae as sistergroup, and again, these worms do not have a bilobed brain. *Paratomella rubra* possesses a dense net of neurites around the statocyst from which dorsolateral tracts extend towards the anterior and posterior, innervating two ring commissures with two dorsolateral neurite bundles extending posteriorly from the posterior ring commissure (Figure 12; [49]).

Finally, the Diopisthoporidae is sistergroup to all other acoels and has been shown to be closest to the inferred ancestor of acoels, although no characters for the nervous system were coded [42]. Using histological serial sections Westblad [54] and Dörjes [46] described the nervous system in *Diopisthoporus psammophilus* and *D. longitubus* as consisting of nervous tissue surrounding the statocyst with two lateral anterior, two dorsal posterior and two ventral posterior neurite bundles emanating from it. Contrary to this Smith and Tyler [48] described a ring-shaped commissure with paired dorsolateral ganglionic lobes immediately posterior to the statocyst and a smaller ring commissure anterior to the statocyst, both rings connected by ventral tracts in *D. gymnopharyngeus* as visualized by electron microscopy (Figure 12; see also Figure 1.5A in [55]). To us it is evident that the ring commissures were overlooked by Westblad and Dörjes, and the report of a SL immunoreactive ring-shaped commissure in *D. longitubus*[47] corroborates this assumption.

Taking the phylogeny of acoels (((Crucimusculata + Prosopharyngida) Paratomellidae) Diopisthoporidae) and the character distribution outlined above into account it is clear that the ground pattern of the acoel nervous system consists of a small number of neurons associated with the statocyst, one to two ring commissures and two to six posterior neurite bundles and a stomatogastric nervous system is absent. This conclusion is further supported by a comparison with the sistergroup of the Acoela, the Nemertodermatida. Only two to four neurite bundles have been found in all described species and ring commissures occur in *Flagellophora apelti*, *Nemertinoides elongatus* and *Nemertoderma westbaldi* (Figure 12; [47,55-58]); a stomatogastric nervous system is also absent [47,56,57].

Remarkably the commissures and neurite bundles in *N. westbladi* and *N. elongatus* are entirely basiepidermal [47,58] and this raises a question regarding the original position of the nervous system in acoelomorphs. *Nemertoderma westbladi* and *N. elongatus* branch off separately at the base of the Nemertodermatida [14] and the only possible outgroups to the Acoelomorpha according to phylogenomic approaches [15,17], cnidarians and xenoturbellids, have intraepithelial plexi. Consequently, in line with observations on many other organ systems [45], the pattern of the nervous system found in nemertodermatids, in which commissures and neurite bundles are positioned at the base of the epidermis, can be taken as the ground pattern for acoelomorphs. It is interesting to note that a trend to displace the commissures and neurite bundles to below the body-wall musculature can be observed in both acoels and nemertodermatids, and that the scant presence of ECM at the base of the epidermis in nemertodermatids and its complete absence in acoels may have facilitated this rearrangement.

The original neuroarchitecture of the Acoelomorpha is characterized by a ring-shaped commissure at the level of the statocyst, a small number of neurite bundles, which are arranged in various positions along the antero-posterior axis without any obvious restriction to the dorsal or ventral side, and a plexus, all aforementioned structures positioned basiepidermally, a small number of neurons associated with the statocyst and the absence of an RFamide-like immunoreactive stomatogastric nervous system. Consequently, in contrast to other proposals [38], the “uracoelomorph” did not have a weakly concentrated nervous system but had a commissural brain, or more specifically a “ring-commissural brain” [47,55-57]. Bilobed brains with one or more “straight” commissures evolved secondarily within the Acoela and the pervasion of such brains by frontal glands and muscles corroborates this – they were simply there before. The selective advantage of an “internalized” brain most likely lies in biomechanical constraints. As shown above the dorsal posterior commissure of *I. pulchra* measures 10 μm in diameter. On average the epidermis of *I. pulchra* measures less than 10 μm in height [29] and consequently the commissure would not fit an intraepidermal position. Furthermore, the shortest connection between two points is a straight line and thus the most economical route with regards to material and energy involves “straight” commissures instead of commissures that follow the circular outline of the body. Once the paired ganglia are submerged below the body-wall musculature they may move closer together to shorten the commissures, eventually to the point at which the brain appears to be unpaired.

One process that could have driven the elaboration of the nervous system in the Crucimusculata is adaptation to a more complex ecology. The majority of taxa at the base of the Acoela are interstitial and supposedly feed on dissolved organic matter [51], whereas in the Crucimusculata many taxa are epibenthic or epiphytic and their diet varies from diatoms to catching active prey like crustaceans, other worms, and even cannibalism can occur [45]. The elaboration of eyes and receptors as well as the introduction of new circuitry to deal with e.g. circadian or tidal rhythms may have appeared concomitant with this ecological transition.

On the other hand we propose that sexual conflict is a driving force for the elaboration of the nervous system. Generally, when looking at the character distribution of sexual traits in a phylogeny of the Acoela there is a trend towards more complexity from “basal” to “divergent” taxa and it has been argued that sexual conflict, the antagonistic co-evolution of male and female sexual traits, nicely accounts for this variation, especially in copulatory organs and sperm ultrastructure [9,45]. To generalize, more complex copulatory organs need a more complex innervation for their function and consequently result in a more complex nervous system. Additionally, complex copulatory organs are related to more complex copulatory behaviour (compare behaviour for mutual exchange in [59,60] with behaviour for hyperdermal transmission [61] and hypodermal injection [60]), and it can be argued that accessory neuronal circuits are necessary for this behaviour.

### Phylogenetic affiliations – evolutionary implications

The nervous system of acoelomorphs comprises three neuroanatomical features that potentially shed light on their phylogenetic affiliations:

1) Arrangement of nerve cords without any dorso-ventral restriction is also found in platyhelminths and ambulacrarians [62-64]. The similarity with platyhelminths is remarkable inasmuch as platyhelminths by all means are nested within the protostomes, which have recently been amended by the inclusion of chaetoghnaths (that develop through deuterostomy), phoronids, brachiopods and bryozoans [65]. All protostomes have the nerve cords, if any are present, restricted to the ventral side, a fact that even suggests the term “Gastroneuralia” to unite them [66-68].

The restriction of nerve cords to one side of the D/V axis is usually regulated through inhibition of *BMP2*/*4* signalling by *chordin*, and interestingly *chordin* seems to be absent in platyhelminths ( [69]; own unpublished observations on [70,71]). In line with many reductions that occurred within this phylum, e.g. of the coelom and anus [72], the *BMP2*/*4* pathway likely has been modulated to allow the circumferential formation of neurite bundles and innervation of the sheet-like body-wall musculature.

Contrary to this acoelomorphs also have circumferentially distributed neurite bundles but *chordin* is present and expressed in a polarized fashion on the ventral side during embryogenesis [73] as in protostomes (excluding Platyhelminthes) and enteropneusts [74]. The neuroarchitecture of the latter, with dorsal and ventral nerve cords, indicates that the primary function of a *BMP2/4-chordin* axis might have been dorsoventral patterning and not the formation of a “polarized” central nervous system [74,75]. However, taking the embryonic expression of *chordin* and the neuroarchitecture of acoelomorphs into account alliances with chordates are impossible and with gastroneuralians very unlikely.

2) A ring-shaped commissure, tract or neurite bundle is present in many taxa throughout the eumetazoans, including in “brainless” animals as in the oral ring of cnidarian polyps and echinoderms. However, in all these cases the alimentary tract passes through the ring-shaped structure in one way or another and the only exception is the “anterior nerve ring” found in enteropneusts [64]. A very detailed map of conserved genes that are involved in anteroposterior (A–P) patterning in *Saccoglossus kowalevskii* shows that specific sets of genes are expressed in front (prosome) or posterior (mesosome) to this circular tract [75,76].

The expression patterns of some orthologue genes of these sets are known from *C. longifissura*, namely *ClSix3/6*, *ClNk2.1*, *ClOtp* and *ClantHox*. They are expressed in distinct subpopulations of neural precursors in the brain primordium during embryonic development and in the region of the dorsal posterior commissure (posterior to the statocyst) and presumptive sensory cells (no data for *ClSix3/6*) in juveniles, *ClantHox* being expressed from the dorsal posterior commissure to the posterior end [19,21].

It is striking that despite direct development such two distinct but different expression patterns occur in a spatially and temporally separate manner and this is reminiscent of hypotheses on a dual origin of brains in protostomes [77] and deuterostomes [78]. Nielsen [77,79] denotes the primary part of the two as the apical and cerebral ganglion, both originating from the larval episphere of present-day protostomes with planktotrophic larvae and the secondary part as the circumblastoporal or ventral brain. Apical ganglion and circumblastoporal brain recapitulate the apical ganglion and circumoral nerve ring of a holopelagic, planktotrophic ancestor, whereas the cerebral ganglia recapitulate the brain of the consecutive ancestor that acquired a benthic lifestyle. Comparisons with this scenario are difficult as a prototroch which marks the limit between epishpere and hyposphere is absent in acoelomorphs as is a stomatogastric nervous system.

Burke [78] divides the nervous system of deuterostomes into a primary animal pole and a secondary axial nervous system and does not corroborate the evolutionary origins of these parts but stresses similarities found in ambulacrarians and chordates.

The presence of such an animal pole nervous system, anterior neuroectoderm (ANE sensu [80]), or “episphere-derived” nervous system in *C. longifissura* can hardly be questioned from three points of view:

Firstly, the embryonic expression pattern of *ClSix3/6*, *ClNk2.1* and *ClOtp* in the brain primordium of *C. longifissura* is most similar to the pattern in the neural tissue of various planktotrophic larvae of bilaterians [77-80], especially *Terebratalia transversa* ( [81]; see also for excellent review).

Secondly, *ClNk2.1*, *ClOtp* and *ClantHox* are expressed posterior to the statocyst in the juvenile whereas a large part of the brain is positioned in front of the statocyst (see Figure 1H in [19] and Figures 3C and D in [18] for SLI in juveniles and Figures 3 and 5C in [8] for SLI and cholinergic nervous system in adults). In *I. pulchra* this part would correspond to the frontal commissure and the anterior lobes and it is quite intriguing that in these structures and the apical organs of various planktotrophic larvae of bilaterians (protostomes and deuterostomes) flask-shaped receptor cells and neurons with SLI occur [38,77-84]. Additionally, in direct-developing hemichordates neurons with SLI develop at the animal pole, disperse into the prosome and seem to persist in adult stages [64].

Last but not least it is intriguing that the ring commissure of *N. westbaldi* comprises an anterior and posterior ring of SLI [47,57] indicating the subdivision at least of the serotonin-like immunoreactive nervous system into an anterior part in front of the statocyst and a posterior part posterior to the statocyst.

With regards to postembryonic development it is evident that the brain of *C. longifissura* is not staggered into an anterior *ClSix3/6* + *ClNk2.1* (protocerebrum-prosome-forebrain) and a middle *ClOtp* domain (deuterocerebrum-mesosome-midbrain) but *ClNk2.1* and *ClOtp* are expressed in an overlapping manner in the area of the dorsal posterior commissure (no data on *ClSix3/6*). Consequently, a forebrain and midbrain boundary as in other bilaterians is absent in acoel(omorph)s and a comparison of the ring-commissure of acoels with the anterior circular tract of enteropneusts on grounds of molecular markers is impossible. Additionally, the tritocerebrum/metasome/hindbrain marker *ClantHox* is expressed from the level of the dorsal posterior commissure to the posterior end but most strongly in paired lateral areas slightly behind the dorsal posterior commissure. In positions identical to these paired lateral areas in the very closely related species *Symsagittifera roscoffensis* groups of neurons extend neurites towards the posterior end [10] and consequently, in respect of neuroarchitecture and expression pattern of *ClantHox*, a correlation with the hindbrain of other bilaterians is striking. However, *ClantHox* is not expressed posteriorly to *ClNk2.1* and *ClOtp* but includes these two domains. Actually the expression pattern found in *C. longifissura* would best be described as nested (see schemes for *ClOtp* and *ClNk2.1* in Figure 2 in [19] and *ClantHox* in Figures 2I, J in [21]). Could this nested pattern be the ancestral state of the axial nervous system? The conservation of staggered and segregated axial nervous systems in bilaterians with different organizations, either diffuse and basiepidermal or centralized and insunk, would favour this assumption. However, investigations on animals with clearly reduced nervous systems, like the horseshoe worm *Phoronopsis harmeri* that develops a transitory paired ventral neurite bundle with serially repeated commissures before metamorphosis [84] are pivotal before derivation from a complex staggered axial brain through reduction can be rejected.

Additionally, more data with a functional basis, on larvae of widespread phylogenetic positions are indispensable. This should also include the expression patterns of Vac and Emx, which are not expressed in staggered domains in the brain of C. longifissura but in the antero-ventral ectoderm and the entire nervous system of late embryos and hatchlings [18], respectively. Concerning the latter it should be emphasised that the ubiquitous expression of ClEmx in C. longifissura does not hint to a posterior growth zone [11] but contrary, in line with observations on the development of the body-wall musculature [32], shows that the nervous system develops through intercalary growth.

3) Absence of a stomatogastric nervous system is remarkable inasmuch as concentrations of neurons around the mouth and oesophagus or pharynx are found throughout the Eumetazoa. Strikingly similar in this respect are only xenoturbellids [5,47,85], worms that live on or in deep marine muds in the North Sea and have been linked with acoelomorphs due to their acoelomate organization, similarities of the multiciliated epidermis [86-89], possession of pulsatile bodies (degenerating epidermal cells that are withdrawn and digested [90,91]) and the lack of an anus, excretory organs and tissues enclosing germ cells [92,93]. Their nervous system consists of a uniform basiepidermal plexus with one type of receptor cell, a statocyst at the anterior end and the absence of a stomatogastric component [85]. The statocyst is so profoundly different from those of the Acoelomorpha that homology must be utterly rejected [94] and even its georeceptive function has been questioned [95].

With regards to the lack of a stomatogastric nervous system in acoelomorphs and xenoturbellids we wish to point out the following:

– Hejnol and Martindale showed expression of the foregut markers *bra* and *gsc* in *C. longifissura* around the mouth and in the entire antero-ventral ectoderm [18]. This indicates that the so-called “catching basket” [96] is homologous to the foregut of other bilaterians. If true, Tyler and Rieger [31] were wrong in suggesting that the complex ventral body-wall musculature evolved to make up for the absence of a pharynx; the pharynx, at least of the Crucimusculata, then, actually would have been extended to the entire ventral surface. In this case the “mouth” would be innervated and we suggest that more research should be conducted on this issue, especially on acoels that have a pharynx and on nemertodermatids.

– The absence of a stomatogastric nervous system in *Xenoturbella bocki* should be taken with a pinch of salt. Raikova et al. [85] reported that the radial muscles and muscles along the gastric cavity display weaker staining than body-wall muscles. No investigation based on histological sections has noted differences between body-wall muscles and inner muscles [92,93,97]. We believe it is possible that the antibodies used to detect neural substances could not penetrate the well-developed subepidermal membrane complex (antibodies are many times larger than the molecule phalloidin) and that experiments should to be conducted on cut specimens before this issue can be settled confidently.

– In cnidarians, as in the majority of bilaterians, the mouth and alimentary cavity or tract respectively, are innervated and show strong FMRFamide-like immunoreactivity in sensory cells as well as the plexus [98]. Consequently the absence of a (stomato)gastric nervous system in (xen)acoelomorphs is a derived feature that can be used as an apomophy of the clade but this loss does not imply any phylogenetic affiliation to other bilaterian clades or a basal position.

Acoelomorphs have many more types of receptor cells, move faster, and are much more versatile than xenoturbellids and last but not least they evidently copulate, all this requiring more integration conducted by the nervous system. The unification of acoelomorphs and xenoturbellids superficially suggests that the stem species of xenacoelomorphs and bilaterians had a simple, uniform plexus. However, we believe that the only safe conclusion is that xenoturbellids are spawners as to our knowledge no animal without any noticeable condensation of the nervous system copulates.

With regard to the pattern of the nervous system of the stem species, care must be taken as xenoturbellids may show reduction in some instances, as in the lack of hemidesmosomes [99], which are clearly present in cnidarians [100,101]. The latter also possess condensations of neurons in every stage of the life cycle, an apical tuft or organ in larvae and at least an oral ring in the medusae and polyps. With regards to life cycle anthozoans are the simplest cnidarians and their larvae have at least two types of receptor cells and the adults even more [102]. To us it is clear that the stem species of bilaterians did not only possess bilateral symmetry and mesoderm but additionally a distinct concentration of neurons at the anterior end or even a brain in the sense of Richter et al. [38]. If we envision this organism to have been able to perceive and react (including feeding) to the environment as well as e.g. planktotrophic larvae of recent bilaterians [103] then it should have had some posteriorly extending neurite bundles as well. Whether this nervous system should be called centralized or not is a difficult matter, as illustrated by the same discussion on this organ in acorn worms [64]. Consequently, this anterior condensation was reduced in xenoturbellids, and in acoelomorphs it was crossed by frontal glands, expanded by a statocyst and adapted further to ecological and sexual constraints by evolving one to three commissures and additional posterior neurite bundles, all of which finally sunk below the body-wall musculature (Figure 12).

### Value of *Isodiametra pulchra* as a model system

Even though the phylogeny of acoelomorphs is by now reasonably well known and the evolution of major characters can be traced satisfyingly [45] a major disadvantage of these animals is the difficulty in culturing them. This applies especially to basal branching species with the exception of *Hofstenia miamia* ( [104], personal communication Mansri Srivastava). This species has conserved many ancestral traits [42] and is amendable to laboratory cultures, but its remarkable size (up to 9 mm) and the possession of pigment may cause other problems.

Even though the internal bilobed brain of *Isodiametra pulchra* is clearly derived investigations on this organ will prove to be auspicious to science. Its small size and the small number of neurons (personal estimation 1k) may allow us to reconstruct the connectome and may even provide an insight to the synaptome [105], enabling us to address various biological questions, such as the general constraints under which a brain works or the conservation of certain circuits through evolution. Among the methods and tools that have been established (ISH, RNAi, ESTs, transcriptome and genome imminent) the establishment of high-pressure freezing in these animals shows great promise for this undertaking [106]. Furthermore, the complex pattern of muscles will be useful in delimiting various regions of the brain, as has been shown in the microturbellarian *Macrostomum lignano*[107].

## Conclusions

*Isodiametra pulchra* possesses a nervous system that comprises a bilobed brain with a dorsal posterior commissure, a frontal ring and tracts, four pairs of longitudinal neurite bundles, as well as a supramuscular and submuscular plexus. There is a highly conserved neuro-muscular system constituted by the statocyst, tracts, classical motor neurons and inner muscles. This neuro-muscular system accounts very nicely for a behaviour that escaped the notice of Tyler and Rieger [31]: it is impossible to turn specimens on their back as they counter-react to all manipulations without delay. Obviously, the direction in which animals move is controlled by the neurons that directly transfer stimuli from the statocyst to the inner muscles. It is remarkable that muscles found to execute quick and strong contractions are pseudostriated ( [31]; own observations) and innervated by FMRFamide-related immunoreactive neurites. These subtypes of muscles also develop through the deployment of different sets of transcription factors (Marta Chiodin, personal communication).

We found that variability among adult specimens is highly correlated with age and wish to stress that even studies on adults must be carried out using specimens with a reliably determined age (e.g. 3 weeks).

The insunk, bilobed brains with two to three commissures of *I. pulchra* and other acoels evolved independently from those found in spiralians and derive from a ring-commissural brain that is genuinely also present in nemertodermatids. This brain is spatially and temporally bipartite, consisting of a *Six3/6*-dependend animal pole nervous system that persists throughout adulthood and an axial nervous system that is not staggered as in other bilaterians but rather nested (terminology of animal pole nervous system and an axial nervous system from Burke [78]).

Most parsimoniously this nervous system stems either from an ancestor with a bipartite brain that was not crossed by the alimentary tract and not staggered into forebrain, midbrain and hindbrain along the A-P axis but into anterior pole and axial nervous system or from an ancestor with a biphasic life cycle and an actively swimming and feeding larva. In the latter case acoelomorphs descended through progenesis and the axial nervous system of the adult ancestor either was primarily nested or the staggered pattern was altered through forestalling its translation to the larval phase.

## Materials and methods

Specimens of *Isodiametra pulchra* and the diatom *Nitzschea curvillineata* (SAG, Göttingen) were kept in f/2 culture medium in a SANYO MLR-350 versatile climate chamber with the temperature set to 18°C and a light/dark regime of 14/10 h. All specimens were anaesthetized with 7.14% magnesium chloride hexahydrate before fixation with freshly made 4% PFA (dissolved in 0.1M PBS at pH 7.5) for histochemistry and immunocytochemistry, or after Eisenmann and Alfert as described in [106] for histology and electron microscopy. Cholinesterases were direct-colored following the protocol of [36]. Immunohistochemistry was conducted as follows after fixation: five washes in PBT (0.1 M PBS with 0.1% Triton-X), blocking specimens and primary antibodies in PBT with 6% NGS (Normal Goat Serum, Invitrogen Corporation, Camarillo, CA) for 1 h with shaking at RT, incubation on shaker o/n at 4°C, five washes with PBT, blocking specimens and secondary antibodies in PBT with 6% NGS for 1 h with shaking at RT, incubation o/n at 4°C with shaking, five washes with PBT, eventual incubation with phalloidin for 1 h at RT followed by three washing steps with PBS, and mounting with FluoromountG (Southern Biotech, Birmingham, AL) or Vectashield (Vector Laboratories, Burlingame, CA), letting the preparations harden o/n at 4°C. The following antibodies and fluorophore-tagged phalloidins were used at the corresponding concentration: polyclonal 5HT produced in rabbit (Sigma, St. Louis, MO) 1:1000; monoclonal 5HT produced in mouse (Abcam, Cambridge UK) 1:10; FMRFamide (DiaSorin, Stillwater, MN) 1:3000; monoclonal tyrosinated tubulin produced in mouse (Sigma, St. Louis, MO) 1:200; Alexa Fluor 488 Rabbit Anti-MouseAlexa (Molecular Probes, Eugene, OR) 1:200; Alexa Fluor 568 Goat Anti-Rabbit (Molecular Probes, Eugene, OR) 1:1000; Alexa Fluor 488 Phalloidin (Molecular Probes, Eugene, OR) and Alexa Fluor 635 Phalloidin (Molecular Probes, Eugene, OR) 1:100. The controls for specificity included omitting the primary antibody and using non-immune serum. Specimens were examined with a Leica TCS SP2 or TCS SPE confocal laser-scanning microscope (Leica Microsystems, Wetzlar, Germany).

Specimens for histological sections and electron microscopy were dehydrated in an acetone series (1 × 50%, 1 × 70%, 1 × 90%, 3 × 100%) after fixation and embedded in EPON 812 epoxy resin (Electron Microscopy Sciences, Hatfield, PA). Serial and single sections with a thickness of 0.5 μm were made using a diamond knife mounted in a Butler trough [108] on a Reichert-Jung Ultracut E. Semithin sections were stained with Richardson’s stain [109], mounted with DePeX (SERVA, Heidelberg, Germany), viewed with a Leica DM 5000B compound microscope (Wetzlar, Germany) and photographed with a Leica DFC 490 digital camera (Wetzlar, Germany). Ultrathin sections were stained with uranyl acetate and lead citrate, and examined with a Zeiss Libra 120 transmission electron microscope.

Images and figures were adjusted and prepared using the programs ImageJ and Photoshop CS. In Figures 4A and 11A, the corners have been coloured black or white, respectively, to prevent contrasting corners and hide dirt. All other images have only been adapted using the level and curve adjustments in Image J or Photoshop CS.

The use of acoel flatworms in the laboratory doesn't raise any ethical issues and therefore Regional or Local Research Ethics Committee approvals are not required.

## Competing interests

The authors declare that they have no competing interests.

## Authors’ contribution

JGA cultured the animals used, designed the project, carried out the experiments, data acquisition and data analysis and drafted and critically revised the manuscript. PM acquired the funding for the materials and microscopes and was involved in drafting and critically revising the manuscript. Both authors read and approved the final manuscript.

## References

[B1] Hyman LH The Invertebrates: Platyhelminthes and Rhynchocoela. The acoelomate Bilateria. Volume 2 1951 McGraw Hill Book Co, New York

[B2] Westblad E Studien über Skandinavische Turbellaria Acoela. 5 Arkiv för Zoologi 1948 41A 7 1 82

[B3] Raikova OI Reuter M Kotikova EA Gustafsson MKS A commissural brain! The pattern of 5-HT immunoreactivity in Acoela (Plathelminthes) [Platyhelminthes] Zoomorphology 1998 118 2 69 77 10.1007/s004350050058

[B4] Reuter M Raikova OI Gustafsson MKS An endocrine brain? The pattern of FMRF-amide immunoreactivity in Acoela (Plathelminthes) Tissue Cell 1998 30 1 57 63 10.1016/S0040-8166(98)80006-2 9569678

[B5] Reuter M Raikova OI Gustafsson MKS Patterns in the nervous and muscle systems in lower flatworms Belgian J Zool 2001 131 47 53

[B6] Reuter M Raikova OI Jondelius U Gustafsson MKS Maule AG Halton DW Organisation of the nervous system in the Acoela: an immunocytochemical study Tissue Cell 2001 33 2 119 128 10.1054/tice.2000.0134 11392663

[B7] Raikova OI Reuter M Gustafsson MKS Maule AG Halton DW Jondelius U Evolution of the nervous system in Paraphanostoma (Acoela) Zool Scripta 2004 33 1 71 88 10.1111/j.1463-6409.2004.00137.x

[B8] Gaerber CW Salvenmoser W Rieger RM Gschwentner R The nervous system of Convolutriloba (Acoela) and its patterning during regeneration after asexual reproduction Zoomorphology 2007 126 2 73 87 10.1007/s00435-007-0039-z

[B9] Achatz JG Hooge M Wallberg A Jondelius U Tyler S Systematic revision of acoels with 9+0 sperm ultrastructure (Convolutida) and the influence of sexual conflict on morphology J Zool Sys Evol Res 2010 48 1 9 32 10.1111/j.1439-0469.2009.00555.x

[B10] Bery A Cardona A Martinez P Hartenstein V Structure of the central nervous system of a juvenile acoel, Symsagittifera roscoffensis Dev Genes Evol 2010 220 3–4 61 76 20549514 10.1007/s00427-010-0328-2 PMC2929339

[B11] Semmler H Chiodin M Bailly X Martinez P Wanninger A Steps towards a centralized nervous system in basal bilaterians: insights from neurogenesis of the acoel Symsagittifera roscoffensis Dev Growth Differ 2010 52 8 701 713 10.1111/j.1440-169X.2010.01207.x 20874714

[B12] Bery A Martinez P Acetylcholinesterase activity in the developing and regenerating nervous system of the acoel Symsagittifera roscoffensis Acta Zool 2011 92 4 383 392 10.1111/j.1463-6395.2010.00472.x

[B13] Ruiz-Trillo I Riutort M Littlewood DTJ Herniou EA Baguñà J Acoel flatworms: earliest extant bilaterian metazoans, not members of Platyhelminthes Science 1999 283 5409 1919 1923 10.1126/science.283.5409.1919 10082465

[B14] Wallberg A Curini-Galletti M Ahmadzadeh A Jondelius U Dismissal of Acoelomorpha: Acoela and Nemertodermatida are separate early bilaterian clades Zool Scripta 2007 36 5 509 523 10.1111/j.1463-6409.2007.00295.x

[B15] Hejnol A Obst M Stamatakis A Ott M Rouse GW Edgecombe GD Martinez M Baguñà J Bailly X Jondelius U Wiens M Müller WEG Seaver E Wheeler WC Martindale MQ Giribet G Dunn CW Assessing the root of bilaterian animals with scalable phylogenomic methods Proc R Soc Lond B Biol Sci 2009 276 1677 4261 4270 10.1098/rspb.2009.0896 PMC2817096 19759036

[B16] Paps J Baguna J Riutort M Bilaterian phylogeny: a broad sampling of 13 nuclear genes provides a new lophotrochozoa phylogeny and supports a paraphyletic basal acoelomorpha Mol Biol Evol 2009 26 10 2397 2406 10.1093/molbev/msp150 19602542

[B17] Philippe H Brinkmann H Copley RR Moroz LL Nakano H Poustka AJ Acoelomorph flatworms are deuterostomes related to Xenoturbella Nature 2011 470 7333 255 258 10.1038/nature09676 21307940 PMC4025995

[B18] Hejnol A Martindale MQ Acoel development supports a simple planula-like urbilaterian Philos Trans R Soc London B Biol Sci 2008 363 1493 1501 10.1098/rstb.2007.2239 18192185 PMC2614228

[B19] Hejnol A Martindale MQ Acoel development indicates the independent evolution of the bilaterian mouth and anus Nature 2008 456 7220 382 386 10.1038/nature07309 18806777

[B20] Sikes JM Bely AE Radical modification of the A-P axis and the evolution of asexual reproduction in Convolutriloba acoels Evol Dev 2008 10 5 619 631 10.1111/j.1525-142X.2008.00276.x 18803779

[B21] Hejnol A Martindale MQ Coordinated spatial and temporal expression of Hox genes during embryogenesis in the acoel Convolutriloba longifissura BMC Biol 2009 7 65 10.1186/1741-7007-7-65 19796382 PMC2761877

[B22] Sikes JM Bely AE Making heads from tails: development of a reversed anterior-posterior axis during budding in an acoel Dev Biol 2010 338 1 86 97 10.1016/j.ydbio.2009.10.033 19878663

[B23] Semmler H Bailly X Wanninger A Myogenesis in the basal bilaterian Symsagittifera roscoffensis (Acoela) Front Zool 2008 5 14 10.1186/1742-9994-5-14 18803837 PMC2562460

[B24] Moreno E Nadal M Baguñà J Martinez P Tracking the origins of the bilaterian Hox patterning system: insights from the acoel flatworm Symsagittifera roscoffensis Evol Dev 2009 11 5 574 581 10.1111/j.1525-142X.2009.00363.x 19754713

[B25] Chiodin M Achatz JG Wanninger A Martinez P Molecular architecture of muscles in an Acoel and its evolutionary implications J Exp Zool B 2011 316B 6 427 439 10.1002/jez.b.21416 PMC3501712 21538843

[B26] De Mulder K Kuales G Pfister D Willems M Egger B Salvenmoser W Thaler M Gorny AK Hrouda M Borgonie G Ladurner P Characterization of the stem cell system of the acoel Isodiametra pulchra BMC Dev Biol 2009 9 69 10.1186/1471-213X-9-69 20017953 PMC2806412

[B27] Moreno E De Mulder K Salvenmoser W Ladurner P Martinez P Inferring the ancestral function of the posterior Hox gene within the bilateria: controlling the maintenance of reproductive structures, the musculature and the nervous system in the acoel flatworm Isodiametra pulchra Evol Dev 2010 12 3 258 266 10.1111/j.1525-142X.2010.00411.x 20565536

[B28] Klauser MD Smith JPS III Tyler S Ultrastructure of the frontal organ in ConvolutaandMacrostomumspp: significance for models of the turbellarian archetype Hydrobiologia 1986 132 47 52 10.1007/BF00046227

[B29] Smith JPS III Bush L Convoluta pulchran. sp. (Turbellaria: Acoela) from the east coast of North America Trans Am Microsc Soc 1991 110 1 12 26 10.2307/3226735

[B30] Chandler RM Thomas MB Smith JPS III The role of shell granules and accessory cells in eggshell formation in Convoluta pulchra (Turbellaria, Acoela) Biol Bull 1992 182 1 54 65 10.2307/1542180 29304701

[B31] Tyler S Rieger RM Functional morphology of musculature in the acoelomate worm, Convoluta pulchra (Plathelminthes) Zoomorphology 1999 119 3 127 141 10.1007/s004350050087

[B32] Ladurner P Rieger R Embryonic muscle development of Convoluta pulchra (Turbellaria - Acoelomorpha, Platyhelminthes) Dev Biol 2000 222 2 359 375 10.1006/dbio.2000.9715 10837125

[B33] Pfistermüller R Tyler S Correlation of fluorescence and electron microscopy of F-actin-containing sensory cells in the epidermis of Convoluta pulchra (Platyhelminthes: Acoela) Acta Zool 2002 83 1 15 24 10.1046/j.1463-6395.2002.00095.x

[B34] Petrov A Hooge M Tyler S Ultrastructure of sperms in Acoela (Acoelomorpha) and its concordance with molecular systematics Invertebr Biol 2004 123 3 183 197

[B35] Petrov A Hooge M Tyler S Comparative morphology of the bursal nozzles in acoels (Acoela, Acoelomorpha) J Morphol 2006 267 5 634 648 10.1002/jmor.10428 16485278

[B36] Karnovsky MJ Roots L Direct-coloring Thiocholine method for Cholinesterases J Histochem Cytochem 1964 12 3 219 222 10.1177/12.3.219 14187330

[B37] Ferrero E A fine structural analysis of the statocyst in Turbellaria Acoela Zool Scripta 1973 2 1 5 16 10.1111/j.1463-6409.1973.tb00793.x

[B38] Richter S Loesel R Purschke G Schmidt-Rhaesa A Scholtz G Stach T Vogt L Wanninger A Brenneis G Döring C Faller S Fritsch M Grobe P Heuer CM Kaul S Møller OS Müller CHG Rieger V Rothe BH Stegner MEJ Harzsch S Invertebrate neurophylogeny: suggested terms and definitions for a neuroanatomical glossary Front Zool 2010 7 29 10.1186/1742-9994-7-29 21062451 PMC2996375

[B39] Ferrero EA Bedini C Ultrastructural aspects of nervous-system and statocyst morphogenesis during embryonic development of Convoluta psammophila (Turbellaria, Acoela) Hydrobiologia 1991 227 131 137 10.1007/BF00027592

[B40] Henry JQ Martindale MQ Boyer BC The unique developmental program of the acoel flatworm, Neochildia fusca Dev Biol 2000 220 2 285 295 10.1006/dbio.2000.9628 10753516

[B41] Raikova OI Reuter M Justine JL Littlewood DTJ, Bray RA Contributions to the phylogeny and systematic of the Acoelomorpha Interrelationships of the Platyhelminthes. Volume 60 2001 Systematics Association Special Volume Series, London 13 23

[B42] Jondelius U Wallberg A Hooge M Raikova OI How the worm got its pharynx: phylogeny, classification and Bayesian assessment of character evolution in Acoela Syst Biol 2011 60 6 845 871 10.1093/sysbio/syr073 21828080

[B43] Tekle YI Raikova OI Ahmadzadeh A Jondelius U Revision of the Childiidae (Acoela), a total evidence approach in reconstructing the phylogeny of acoels with reversed muscle layers J Zool Sys Evol Res 2005 43 1 72 90 10.1111/j.1439-0469.2004.00293.x

[B44] Bedini C Lanfranchi A The central and peripheral nervous system of Acoela (Plathelminthes). An electron microscopical study Acta Zool 1991 72 2 101 106 10.1111/j.1463-6395.1991.tb00322.x

[B45] Achatz JG Chiodin M Salvenmoser W Tyler S Martinez P The Acoela: on their kind and kinships, especially with nemertodermatids and xenoturbellids (Bilateria incertae sedis) Org Div Evol 2012 10.1007/s13127-012-0112-4 PMC3789126 24098090

[B46] Dörjes J Die Acoela (Turbellaria) der Deutschen Nordseekste und ein neues System der Ordnung Z Zool Syst Evolutionsforsch 1968 6 56 452

[B47] Raikova OI Reuter M Gustafsson MKS Maule AG Halton DW Jondelius U Basiepidermal nervous system in Nemertoderma westbladi (Nemertodermatida): GYIRFamide immunoreactivity Zoology 2004 107 1 75 86 10.1016/j.zool.2003.12.002 16351929

[B48] Smith JPS Tyler S Fine structure and evolutionary implications of the frontal organ in Turbellaria Acoela. I. Diopisthoporus gymnopharyngeus n. sp Zool Scr 1985 14 91 102 10.1111/j.1463-6409.1985.tb00180.x

[B49] Crezée M Paratomella rubra Rieger and Ott, an amphiatlantic acoel turbellarian Cah Biol Mar 1978 19 1 1 9

[B50] Correa DD Two new marine Turbellaria from Florida Bull Mar Sci Gulf Carrib 1960 10 208 216

[B51] Crezée M Monograph of the Solenofilomorphidae (Turbellaria: Acoela) Internationale Revue der Gesamten Hydrobiologie 1975 60 6 769 845 10.1002/iroh.19750600604

[B52] Steinböck O Die Hofsteniiden (Turbellaria Acoela). Grundsätzliches zur Evolution der Turbellarien Z Zool Syst Evolutionsforsch 1966 4 58 195

[B53] Bock S Eine neue Turbellariengattung aus Japan Uppsal Universitets Arsskr Mat Och Nat 1923 1 1 52

[B54] Westbald E Studien über skandinavische Turbellaria Acoela. I Arkiv för Zoologi 1940 32A 20 1 28

[B55] Tyler S Littlewood DTJ, Bray RA The early worm: origins and relationships of the lower flatworms Interrelationships of the Platyhelminthes. Volume 60 2001 Systematics Association Special Volume Series, London 3 12

[B56] Westbald E Die Turbellarien-Gattung Nemertoderma Steinböck Acta Soc Fauna Flora Fennica 1937 60 45 89

[B57] Raikova OI Reuter M Jondelius U Gustafsson MKS The brain of the Nemertodermatida (Platyhelminthes) as revealed by anti-5HT and anti-FMRFamide immunostainings Tissue Cell 2000 32 5 358 365 10.1054/tice.2000.0121 11201275

[B58] Riser NW Nemertinoides elongatus gen. n., sp. n. (Turbellaria: Nemertodermatida) from coarse sand beaches of the western north Atlantic Proc Helminthol Soc Wash 1987 54 1 60 67

[B59] Costello HM Costello DP Copulation in the Acoelous Turbellarian Polychoerus carmelensis Biol Bull 1938 75 85 98 10.2307/1537675

[B60] Apelt G Fortpflanzungsbiologie, Entwicklungszyklen und vergleichende Frühentwicklung acoeler Turbellarien Mar Biol 1969 4 267 325

[B61] Bush L Biology of Neochildia fusca n. gen. n. sp. from the Northeastern Coast of United States (Platyhelminthes-Turbellaria) Biol Bull 1975 148 1 35 48 10.2307/1540648 1115811

[B62] Rieger RM Tyler S Smith JPS III Rieger GE Harrison W, Bogitsh BJ Platyhelminthes: Turbellaria Microscopic Anatomy of Invertebrates. Volume 3. Platyhelminthes and Nemertinea 1991 Wiley-Liss Inc, New York 7 140

[B63] Knight-Jones E On the nervous system of Saccoglossus cambriensis (Enteropneusta) Phil Trans R Soc B Biol Sci 1952 236 315 354 10.1098/rstb.1952.0004

[B64] Nomaksteinsky M Röttinger E Dufur HD Chettouh Z Lowe CJ Martindale MQ Brunet JF Centralization of the Deuterostome nervous system Predates Chordates Curr Biol 2009 19 1 6 10.1016/j.cub.2008.11.058 19559615

[B65] Edgecombe GD Giribet G Dunn CW Hejnol A Kristensen RM Neves RC Rouse GW Worsaae K Sørensen MV Higher-level metazoan relationships: recent progress and remaining questions Org Divers Evol 2011 11 151 172 10.1007/s13127-011-0044-4

[B66] Hatschek B Lehrbuch der Zoologie, 1. Lieferung 1888 Gustav Fischer, Jena

[B67] Schimkewitsch W Versuch einer Klassiffikation des Tierreichs Biologisches Zentralblatt 1891 11 291 295

[B68] Ulrich W Vorschläge zu einer Revision der Grosseinteilung des Tierreiches Verhandlungen der Deutschen Zoologischen Gesellschaft 1951 1950 244 271

[B69] Molina MD Neto A Maeso I Gómez-Skarmeta JL Saló E Cebrià F Noggin and noggin-like genes control dorsoventral axis regeneration in planarians Curr Biol 2011 21 4 300 305 10.1016/j.cub.2011.01.016 21295481

[B70] National Center for Biotechnology Information http://ncbi.nlm.nih.gov

[B71] Macrostomum lignano genome initiativewww.macgenome.org

[B72] Balavoine G Are Platyhelminthes Coelomates without a Coelom? An argument based on the evolution of Hox Genes Amer Zool 1998 38 843 858

[B73] Hrouda M Molecular Analysis of the Evolution of Bilaterian Body Axes: Wnt and Bmp-signalling inIsodiametra pulchraandMacrostomum lignano(Acoelomorpha, Macrostomorpha; Platyhelminthes) PhD thesis 2007 University of Innsbruck, Department of Evolutionary Developmental Biology,

[B74] Lowe CJ Terasaki M Wu M Freeman RM Runft L Kwan K Haigo S Aronowicz Lander E Gruber C Smith M Kirschner M Gerhart J Dorsoventral patterning in Hemichordates: insights into early Chordate evolution PLoS Biol 2006 4 9 e291 10.1371/journal.pbio.0040291 16933975 PMC1551926

[B75] Lowe CJ Molecular genetic insight into deuterostome evolution from the direct-developing hemichordate Saccoglossus kowalevskii Phil Trans R Soc B Biol Sci 2008 363 1569 1578 10.1098/rstb.2007.2247 PMC2615823 18192177

[B76] Lowe CJ Wu M Salic A Evans L Lander E Stange-Thomann N Gruber CE Gerhart J Kirschner M Anteroposterior patterning in Hemichordates and the origins of the Chordate nervous system Cell 2003 113 853 865 10.1016/S0092-8674(03)00469-0 12837244

[B77] Nielsen C Larval and adult brains Evol Dev 2005 7 5 483 489 10.1111/j.1525-142X.2005.05051.x 16174040

[B78] Burke RD Deuterostome neuroanatomy and the body plan paradox Evol Dev 2011 13 1 110 115 10.1111/j.1525-142X.2010.00460.x 21210947

[B79] Nielsen C How to make a protostome Invertebr Syst 2012 26 25 40 10.1071/IS11041

[B80] Angerer LM Yaguchi S Angerer RC Burke RD The evolution of nervous system patterning: insights from sea urchin development Development 2011 138 3613 3623 10.1242/dev.058172 21828090 PMC3152920

[B81] Hay-Schmidt A The evolution of the serotonergic nervous system Proc R Soc Lond B Biol Sci 2000 267 1071 1079 10.1098/rspb.2000.1111 PMC1690648 10885511

[B82] Santagata S Resh C Hejnol A Martindale MQ Passamaneck YJ Development of the larval anterior neurogenic domains of Terebratalia transversa (Brachiopoda) provides insights into the diversification of larval apical organs and the spiralian nervous system Evodevo 2012 3 3 10.1186/2041-9139-3-3 22273002 PMC3314550

[B83] Nakajima Y Humphreys T Kaneko T Tagawa K Development and neural organization of the tornaria larva of the Hawaiian hemichordate, Ptychodera flava Zool Sci 2004 21 69 78 10.2108/0289-0003(2004)21[69:DANOOT]2.0.CO;2 14745106

[B84] Temereva E Wanninger A Development of the nervous system in Phoronopsis harmeri (Lophotrochozoa, Phoronida) reveals both deuterostome- and trochozoan-like features BMC Evol Biol 2012 12 121 10.1186/1471-2148-12-121 22827441 PMC3441923

[B85] Raikova OI Reuter M Jondelius U Gustafsson MKS An immunocytochemical and ultrastructural study of the nervous and muscular systems of Xenoturbella westbladi (Bilateria inc. sed.) Zoomorphology 2000 120 2 107 118 10.1007/s004350000028

[B86] Pedersen KJ Pedersen LR Fine ultrastructural observations on the extracellular matrix (ECM) of Xenoturbella bocki Westblad, 1949 Acta Zool 1986 67 2 103 113 10.1111/j.1463-6395.1986.tb00854.x

[B87] Franzén A Afzelius BA The ciliated epidermis of Xenoturbella bocki (Platyhelminthes, Xenoturbellida) with some Phylogenetic considerations Zool Scr 1987 16 1 9 17 10.1111/j.1463-6409.1987.tb00046.x

[B88] Pedersen KJ Pedersen LR Ultrastructural observations on the epidermis of Xenoturbella bocki Westblad, 1949; with a discussion of epidermal cytoplasmic filament systems of invertebrates Acta Zool 1988 69 4 231 246 10.1111/j.1463-6395.1988.tb00920.x

[B89] Lundin K The epidermal ciliary rootlets of Xenoturbella bocki (Xenoturbellida) revisited: new support for a possible kinship with the Acoelomorpha (Platyhelminthes) Zool Scr 1998 27 3 263 270 10.1111/j.1463-6409.1998.tb00440.x

[B90] Lundin K Hendelberg J Degenerating epidermal bodies ("pulsatile bodies") in Meara stichopi (Plathelminthes, Nemertodermatida) Zoomorphology 1996 116 1 1 5 10.1007/BF02526924

[B91] Lundin K Degenerating epidermal cells in Xenoturbella bocki (phylum uncertain), Nemertodermatida and Acoela (Platyhelminthes) Belgian J Zool 2001 131 153 157

[B92] Westblad E Xenoturbella bocki n. g., n. sp. a peculiar, primitive turbellarian type Arkiv för Zoologi 1949 1 11 29

[B93] Hyman LH The Invertebrates: Smaller Coelomate Groups (Vol. 5 1959 McGraw Hill Book Co, New York

[B94] Ehlers U Comparative morphology of statocycts in the Plathelminthes and the Xenoturbellida Hydrobiologia 1991 227 263 271 10.1007/BF00027611

[B95] Israelson O Ultrastructural aspects of the ‘statocyst’ of Xenoturbella (Deuterostomia) cast doubt on its function as a georeceptor Tissue Cell 2007 39 171 177 10.1016/j.tice.2007.03.002 17434196

[B96] Shannon T Achatz JG Convolutriloba macropygasp. nov., an uncommonly fecund acoel (Acoelomorpha) discovered in tropical aquaria Zootaxa 2007 1525 1 17

[B97] Reisinger E Was ist Xenoturbella? Zeitschrift fuer Wissenschaftliche Zoologie 1960 164 188 198

[B98] Grimmelikhuijzen CJP FMRFamide immunoreactivity is generally occuring in the nervous system of Coelenterates Histochemistry 1983 78 361 381 10.1007/BF00496623 6136494

[B99] Ehlers U Sopott-Ehlers B Ultrastructure of the subepidermal musculature of Xenoturbella bocki, the adelphotaxon of the Bilateria Zoomorphology 1997 117 71 79 10.1007/s004350050032

[B100] Chapman JA Kirkness EF Simakov O Hampson SE Mitros T Weinmaier T Rattei T Balasubramanian G Borman J Busam D Disbennett K Pfannkoch C Sumin N Sutton GG Viswanathan LD Walenz B Goodstein DM Hellsten U Kawashima T Prochnik SE Putnam NH Shu S Blumberg B Dana CE Gee L Kibler DF Law L Lindgrens D Martinez DE Peng J Wigge PA Bertulat B The dynamic genome of Hydra Nature 2010 464 592 596 10.1038/nature08830 20228792 PMC4479502

[B101] Holstein TW Hess MW Salvenmoser W Müller-Reichert T Preparation techniques for transmission electron microscopy of Hydra Methods in Cell Biology. Volume 96 2010 Academic, Heidelberg 286 302 10.1016/S0091-679X(10)96013-5 20869528

[B102] Fautin DG Mariscal RN Harrison FW, Westfall JA Cnidaria: anthozoa Microscopic Anatomy of Invertebrates. Volume 2. Placozoa, Porifera, Cnidaria, and Ctenophora 1990 Wiley-Liss, New York 267 358

[B103] Dautov SSH Nezlin LP Nervous system of the Tornaria Larva (Hemichordata: Enteropneusta). A histochemical and ultrastructural study Biol Bull 1992 183 463 475 10.2307/1542023 29300513

[B104] Hooge M Wallberg A Todt C Maloy A Jondelius U Tyler S A revision of the systematics of panther worms (Hofstenia spp., Acoela), with notes on color variation and genetic variation within the genus Hydrobiologia 2007 592 1 439 454 10.1007/s10750-007-0789-0

[B105] DeFelipe J From the Connectome to the Synaptome: an epic love story Science 2010 330 1198 1201 10.1126/science.1193378 21109663

[B106] Salvenmoser W Egger B Achatz JG Ladurner P Hess MW Müller-Reichert T Electron microscopy of flatworms: standart and Cryo-preparation methods Methods in Cell Biology. Volume 96 2010 Academic, Heidelberg 307 330 10.1016/S0091-679X(10)96014-7 20869529

[B107] Morris J Cardona A De Miguel-Bonet MDM Hartenstein V Neurobiology of the basal platyhelminth Macrostomum lignano: map and digital 3D model of the juvenile brain neuropile Dev Genes Evol 2007 217 8 569 584 10.1007/s00427-007-0166-z 17611771

[B108] Butler JK Methods for improved light-microscope microtomy Stain Technology 1979 54 2 53 69 494331 10.3109/10520297909112636

[B109] Richardson KC Jarett L Finke EH Embedding in epoxy resins for ultrathin sectioning in electron microscopy Stain Technol 1960 35 6 313 323 13741297 10.3109/10520296009114754

